# Deletion of gmfA induces keratocyte‐like migration in *Dictyostelium*

**DOI:** 10.1002/2211-5463.13339

**Published:** 2021-12-12

**Authors:** Koushiro Fujimoto, Kentaro Nakano, Hidekazu Kuwayama, Shigehiko Yumura

**Affiliations:** ^1^ Graduate School of Sciences and Technology for Innovation Yamaguchi University Japan; ^2^ Graduate School of Life and Environmental Sciences University of Tsukuba Japan

**Keywords:** actin, Arp2/3 complex, cell division, cell migration, chemotaxis, pseudopods

## Abstract

Glia maturation factor (GMF) has been established as an inactivating factor of the actin‐related protein 2/3 (Arp2/3) complex, which regulates actin assembly. Regulation of actin assembly and reorganization is crucial for various cellular events, such as cell migration, cell division, and development. Here, to examine the roles of ADF‐H domain‐containing protein (also known as glia maturation factor; GmfA), the product of a single GMF homologous gene in *Dictyostelium*, gmfA‐null cells were generated. They had moderate defects in cell growth and cytokinesis. Interestingly, they showed a keratocyte‐like fan shape with a broader pseudopod, where Arp3 accumulated at higher levels than in wild‐type cells. They migrated with higher persistence, but their velocities were comparable to those of wild‐type cells. The polar pseudopods during cell division were also broader than those in wild‐type cells. However, GmfA did not localize at the pseudopods in migrating cells or the polar pseudopods in dividing cells. Adhesions of mutant cells to the substratum were much stronger than that of wild‐type cells. Although the mutant cells showed chemotaxis comparable to that of wild‐type cells, they formed disconnected streams during the aggregation stage; however, they finally formed normal fruiting bodies. These results suggest that GmfA plays a crucial role in cell migration.

## Graphical abstract

Glia maturation factor (GMF) has been established as an inactivating factor of the Arp2/3 complex. Here, *gmfA* knock‐out mutant *Dictyostelium* showed a keratocyte‐like fan shape with a broader pseudopod, where Arp3 accumulated at higher levels than in wild‐type cells. The mutant cells migrated with higher persistence. These results suggest that GmfA plays a crucial role in cell migration.

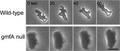

**Abbreviations**
ADF: actin‐depolymerizing factor
Arp2/3: actin‐related protein 2/3
GFP: green fluorescent protein
GMF: Glia maturation factor
MSI: mitosis stage index
PCR: polymerase chain reaction

Glia maturation factor (GMF) is a ˜ 17 kDa protein that is widely conserved from yeast to mammals [1]. GMF was first discovered as an inducer of neural differentiation [2, 3]. Mammalian cells have two isoforms, GMFβ and GMFγ, both of which colocalize with actin filaments and regulate them [4, 5]. The structure of GMF has been solved [6, 7], revealing it to be a bona fide member of the actin‐depolymerizing factor homology (ADF‐H) family that includes ADF/cofilin, twinfilin, actin‐binding protein 1 (Abp1)/drebrin, and coactosin. All known ADF‐H proteins bind to either actin and/or Arp2/3 complex and have conserved roles in actin cytoskeleton remodeling [8]. Moreover, GMF has been established as an inactivating factor of the Arp2/3 complex in yeast and *Drosophila* [9, 10, 11].

The Arp2/3 complex is involved in cell migration and cell division by nucleating and branching actin filaments [12]. The activators of the Arp2/3 complex include Wiskott–Aldrich syndrome protein (WASP), suppressor of cAMP receptor (SCAR), and Wiskott–Aldrich syndrome protein and SCAR homolog (WASH), whose regulatory mechanisms have been intensively studied [13, 14, 15, 16].

On the other hand, there is limited information available regarding the inactivation of the Arp2/3 complex. Actin filaments are stable in the presence of the Arp2/3 complex *in vitro* and do not depolymerize for several minutes; however, these filaments repeatedly polymerize and depolymerize within a few seconds within a cell [17, 18, 19, 20]. Therefore, the inactivating factor of the Arp2/3 complex should cope with the rapid dynamics of the actin structures in a living cell. Cofilin, coronin, and GMF are candidates for the inactivating factors. Cofilin is known to bind to actin filaments and participate in their cleavage and depolymerization [21]. Deletion mutants of the gene encoding this protein in yeast, nematodes, *Drosophila*, and *Dictyostelium* are lethal [22]. Coronin binds to both actin filaments and Arp2/3 complex and suppresses actin filament assembly [23, 24, 25].

Glia maturation factor has a domain homologous to cofilin and is classified as a cofilin‐related protein [1]. However, unlike cofilin and coronin, GMF does not directly bind to actin filaments, but inhibits actin nucleation and cuts the branched actin filaments by binding to the Arp2/3 complex [1]. GMFs in yeast, *Drosophila*, and *Dictyostelium* promote the disassembly of actin filaments *in vitro* [9, 10, 11]. However, there is relatively little information available regarding the *in vivo* role of GMF in regulating actin dynamics; nonetheless, studies on mammalian cultured cells have suggested that GMF associates with the leading edges of migrating cells and contributes to cell migration [5, 11, 26, 27].

Here, we investigated the functions of GMF in *Dictyostelium*, which is a model organism for studying cell migration, cell division, and development. *Dictyostelium* has a single GMF homolog, *gmfA* [10]. To examine the role of gmfA, gmfA‐null cells were generated. They showed defects in cell growth and cytokinesis. They had a keratocyte‐like morphology with a broader fan‐like pseudopod and migrated with high persistency. These pseudopods contained higher levels of Arp2/3 complex than the wild‐type cells. In addition, they adhered to the substratum more strongly than wild‐type cells. In the aggregation stage, gmfA‐null cells frequently lost their connections toward the center of aggregation during stream formation, but finally formed normal fruiting bodies. We have also discussed the role of gmfA at the molecular level.

## Results

### GmfA‐null cells have defects in cell growth and cytokinesis

**Fig. 1:**
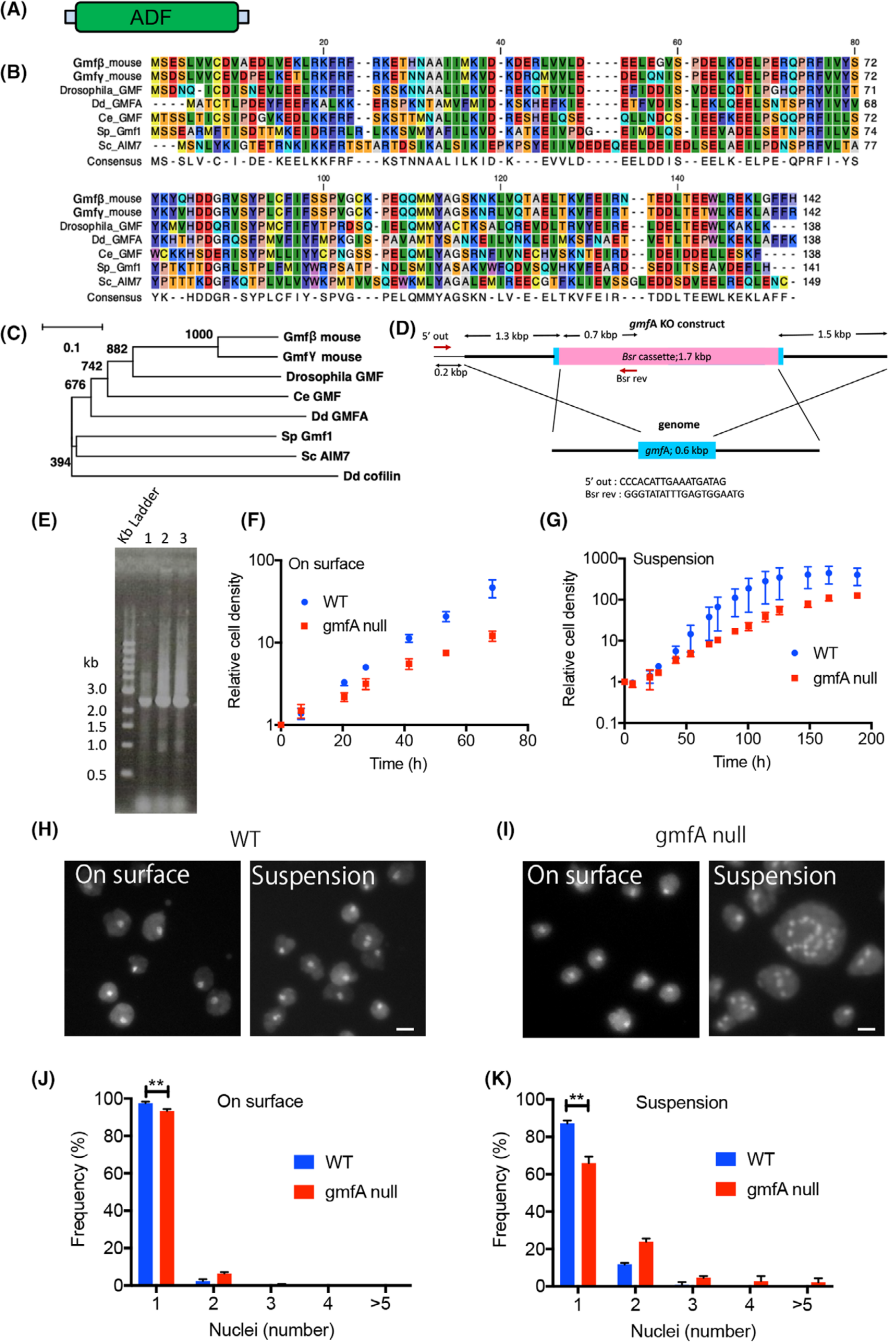
GmfA‐null cells have defects in cell growth and cytokinesis. (A) GmfA is composed of 138 amino acids and has a single ADF/cofilin domain (ADF). (B) A comparison of amino acid sequences among mouse GMFs β and γ, *Drosophila melanogaster* GMF, *Caenorhabditis elegans* GMF, *Schizosaccharomyces pombe* Gmf1, *Saccharomyces cerevisiae* Aim7, and *Dictyostelium discoideum* gmfA. (C) A phylogenetic tree for these GMFs and *Dictyostelium* cofilin. (D) The design of the knockout construct. © Genomic PCR of three independent clones. Genomic PCR was conducted using primer 5′ out and Bsr rev (panel D). The 2.2 kb bands correspond to the gene disrupted allele. The clone No. 1 was used for the experiments. (F, G) Growth curve of wild‐type and gmfA‐null cells in a plastic dish and shaking culture, respectively. Data are represented as the mean ± SD (3 experiments). (H, I) Typical fluorescence images of DAPI‐stained wild‐type and gmfA‐null cells in a plastic dish (On surface) and shaking culture (Suspension), respectively. Scale bars = 10 µm. (J, K) Frequencies of multinucleation of wild‐type and gmfA‐null cells in a plastic dish and shaking culture, respectively. More than 500 cells were counted from individual cell lines. Data are represented as the mean ± SD. **P < 0.001, Student’s *t*‐test.

*Dictyostelium* has a single GMF homolog gene, *gmfA*; its product has a molecular weight of 16 kDa and consists of 138 amino acids. GmfA has a single ADF/cofilin domain (Fig. 1A). Figure 1B shows a comparison of the amino acid sequences of GMFs found in mouse, fruit fly, nematode, fission yeast, budding yeast, and *Dictyostelium*. Figure 1C shows a phylogenetic tree for these GMFs and cofilins in *Dictyostelium*, indicating that gmfA is more closely related to these GMFs than cofilin and that it is more closely related to mouse GMFs than yeast GMFs.

We have previously shown that isolated gmfA from *Dictyostelium* cells suppresses the activation of actin assembly by Arp2/3 complex *in vitro* [10]. In the present study, we generated knockout mutants by homologous recombination using the knockout construct depicted in Fig. 1D. Disruption of the *gmfA* gene in three independent clones was confirmed by genomic PCR using two primers (Fig. 1E). Figure 1F,G show the growth curves in plastic dishes and shaking cultures, respectively. The doubling times of gmfA‐null cells (18.0 ± 1.2 h on surface and 19.6 ± 2.1 h in suspension condition, *n* = 3) were much longer than those of wild‐type cells (11.1 ± 0.7 h on surface and 9.6 ± 2.5 h in suspension condition, *n* = 3), suggesting that these mutants show defective growth in both the conditions.

To observe their nuclei, cells were fixed and stained with DAPI after 72 h of culture in both the conditions (Fig. 1H,I). The mutant cells were observed to be significantly multinucleated, especially under the suspension condition (Fig. 1J,K). Thus, gmfA‐null cells showed a defect in cytokinesis, although it did not seem to be severe on the surface of a substrate.

### GmfA‐null cells have a keratocyte‐like shape with a broader pseudopod

**Fig. 2:**
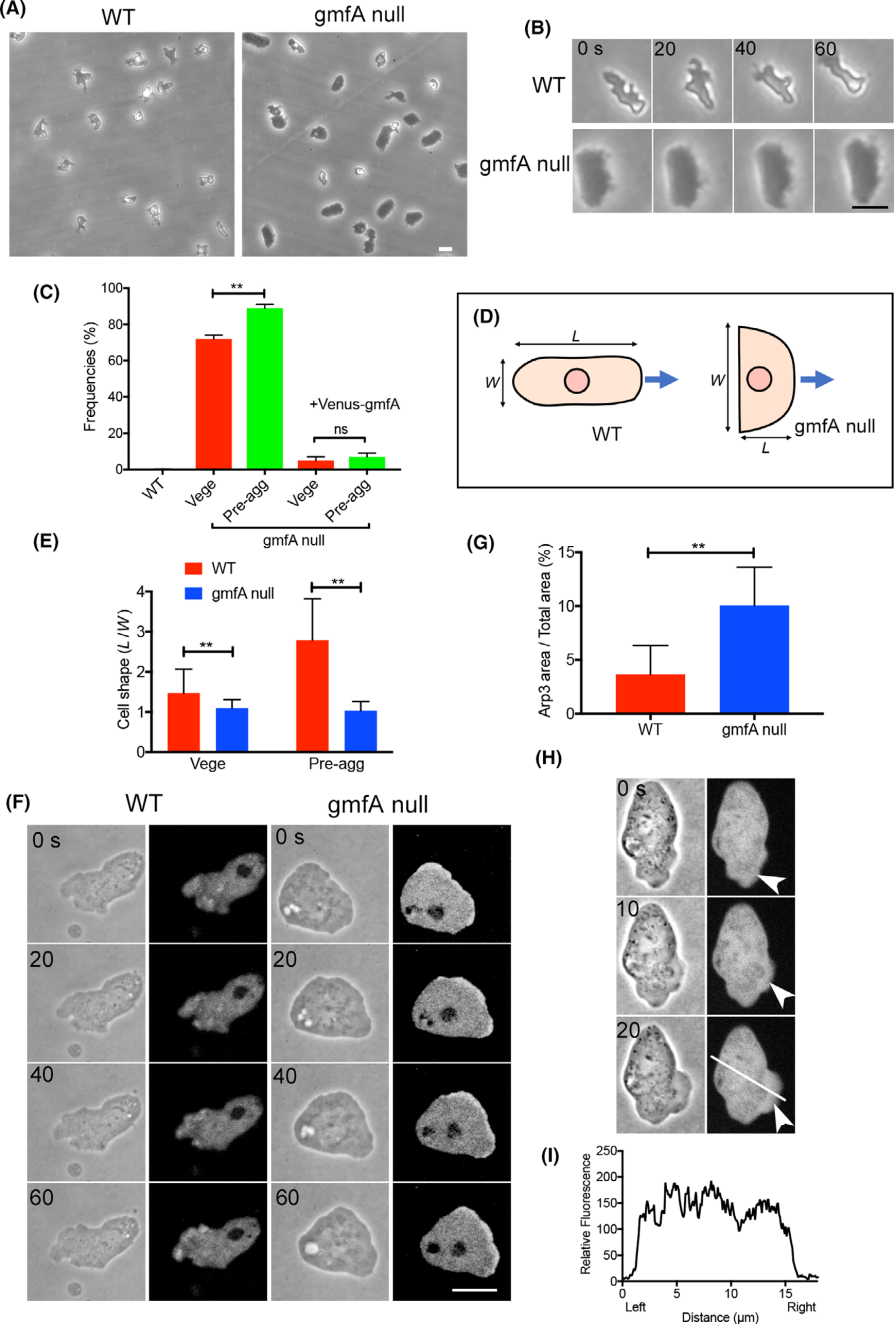
GmfA‐null cells have a keratocyte‐like shape with a broader pseudopod. (A) Typical phase‐contrast images of migrating wild‐type and gmfA‐null cells. Scale bar = 10 µm. (B) A typical time course of phase‐contrast images of a migrating gmfA‐null cell. Scale bar = 10 µm. (C) Frequencies of keratocyte‐like cells in vegetative and pre‐aggregation stages. Those of gmfA‐null cells expressing Venus‐gmfA were also plotted. Data are represented as the mean ± SD. **P < 0.001; ns, not significant, *P* > 0.05 (*n* > 200 for each, Student’s *t*‐test). (D) Cell shape index (*L*/*W*) was calculated by dividing the cell length in the migration direction (*L*) by the width (*W*). The cell shape index of gmfA‐null cells was examined on keratocyte‐like cells. © A comparison of cell shape index between vegetative and pre‐aggregation stages in wild‐type and keratocyte‐like mutant cells. Data are represented as the mean ± SD (*n* > 50 for each). **P < 0.001, Student’s *t*‐test. (F) Typical time courses of phase‐contrast and fluorescence images of migrating cells expressing GFP‐Arp3 in wild‐type and gmfA‐null cells, respectively. Scale bar = 10 µm. (G) The relative areas of pseudopods (containing GFP‐Arp3) in wild‐type and gmfA‐null cells. Data are represented as the mean ± SD (*n* > 50 for each). **P < 0.001, Student’s *t*‐test. (H) A typical time course of phase‐contrast and fluorescence images of a migrating gmfA‐null cell expressing Venus‐gmfA. Arrowheads indicate pseudopods at the leading edge. (I) A typical line scanning of fluorescence intensities along the white line in panel H.

Figure 2A shows phase‐contrast microscopy images of wild‐type and gmfA‐null cells. GmfA‐null cells appeared much darker than the wild‐type cells. Although wild‐type cells migrated in the direction of their long axis with a frequently changing shape, the mutant cells migrated in the direction of their short axis with a fan‐like shape, resembling fish epidermal keratocyte migration (Fig. 2B). Although wild‐type cells extended multiple pseudopods with repeated extension and retraction, gmfA‐null cells constantly extended a single broader pseudopod without retraction. Interestingly, some of the mutant cells transiently converted to a normal shape comparable to that of wild‐type cells and returned to keratocyte‐like (Movies S1 and S2). They could retract pseudopods when their shapes were normal. Figure 2C shows the frequencies of the keratocyte‐like cells including the transiently converted cells in 30‐min movies. The frequencies were approximately 72% of the population in vegetative cells and 89% of the population in the pre‐aggregation stage (6 h after starvation). Incidentally, no wild‐type cells showed keratocyte‐like shape (Fig. 2C). To quantify the cell shape, cell shape index (*L*/*W*) was calculated by dividing the cell length in the migration direction (*L*) by the width (*W*), as depicted in Fig. 2D. Figure 2E shows a comparison of the cell shape index between vegetative and pre‐aggregation stages in wild‐type and mutant cells, respectively. Wild‐type cells elongated in the migration direction in the pre‐aggregation stage, but the mutant cells maintained keratocyte‐like shape.

Figure 2F shows typical time courses of fluorescence images of migrating cells expressing GFP‐Arp3 in wild‐type and gmfA‐null cells. GFP‐Arp3 localized at the pseudopods in the wild‐type cells (Fig. 2F, left panel), which was consistent with previous reports [28, 29]. GmfA‐null cells had much larger and broader pseudopods containing Arp3 (Fig. 2F, right panel) than wild‐type cells (left panel). Figure 2G shows the relative areas of pseudopods (containing GFP‐Arp3) in wild‐type and gmfA‐null cells, showing that gmfA‐null cells had a 2.8 times larger pseudopod area than that observed for wild‐type cells. It is plausible that the deletion of the inactivation of Arp2/3 activities enhances actin assembly, thereby resulting in broader pseudopods.

Figure 2H shows the typical time courses of fluorescence images of migrating cells expressing Venus‐gmfA (left). Movie S3 also shows a typical time‐lapse movie of another cell. Here, Venus‐gmfA was expressed in gmfA‐null cells and the frequencies of keratocyte‐shaped cells were greatly reduced (Fig. 2C), indicating that Venus‐gmfA complements the defect of null cells. Venus‐gmfA slightly appeared to preferentially localize at the pseudopods (arrowheads), but a typical line scanning of fluorescence intensities (Fig. 2I) suggested that it did not localize, especially at the pseudopods.

### GmfA‐null cells migrate with a higher persistency and adhere strongly to the substratum

**Fig. 3:**
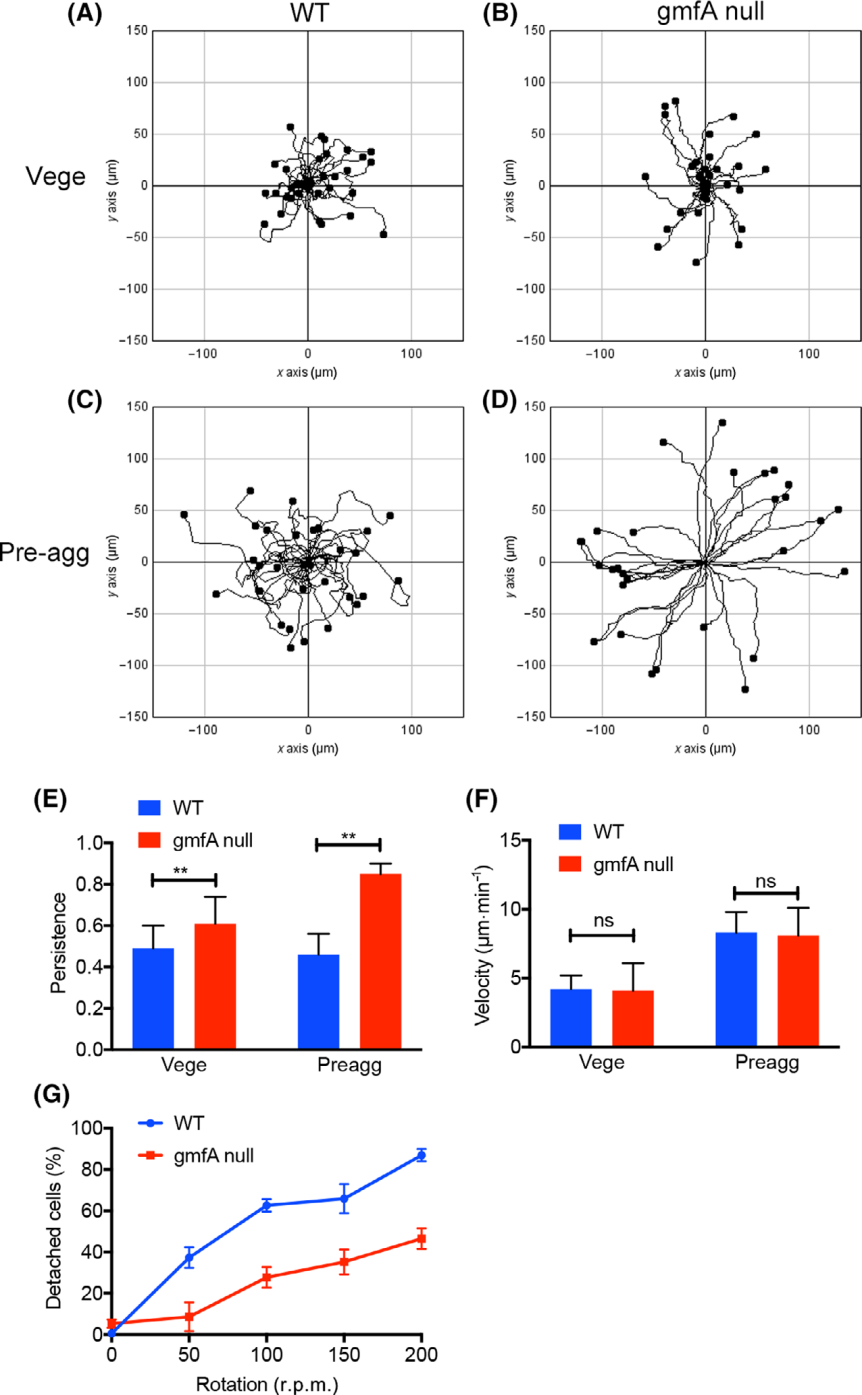
GmfA‐null cells migrate with a higher persistency and adhere strongly to the substratum. (A–D) Results of cell tracking of wild‐type and gmfA‐null cells in vegetative and pre‐aggregation stages, respectively. © A comparison of the persistence of cell migration in wild‐type and gmfA‐null cells in vegetative and pre‐aggregation stages, respectively. Data are represented as the mean ± SD (*n* > 50 for each). **P < 0.001, Student’s *t*‐test. (F) Average velocities of wild‐type and gmfA‐null cells in vegetative and pre‐aggregation stages, respectively. Data are represented as the mean ± SD (*n* > 50 for each). Ns, not significant, *P* > 0.05, Student’s *t*‐test. (G) Results of adhesion assay. Thirty minutes after cells were attached to the bottom surface of the culture dish, the detached cells were counted 10 min after shaking by a reciprocal shaker at various speeds (r.p.m.). Data are represented as the mean ± SD (3 experiments).

Keratocytes tend to move without largely changing their direction (therefore, with a higher persistence). Figures 3A–D show the results of tracking of wild‐type and gmfA‐null cells in vegetative and pre‐aggregation stages, respectively. Figure 3E shows their persistence indices, suggesting that gmfA‐null cells migrated with a higher persistence than the wild‐type cells. However, the average velocities of wild‐type and gmfA‐null cells were not significantly different in either the vegetative or the pre‐aggregation stage (Fig. 3F).

Since gmfA‐null cells appeared darker than the wild‐type cells under phase‐contrast microscopy (Fig. 2A), their adhesion to the substratum was thought to have affected their cell shape. Hence, we next performed an adhesion assay. In this assay, 30 min after the cells were attached to the bottom surface of the culture dish; the detached cells were counted 10 min after shaking by a reciprocal shaker at various speeds (r.p.m.). As shown in Fig. 3G, gmfA‐null cells were found to adhere much more strongly to the substratum than the wild‐type cells.

### Dividing gmfA‐null cells have broader polar pseudopods

**Fig. 4:**
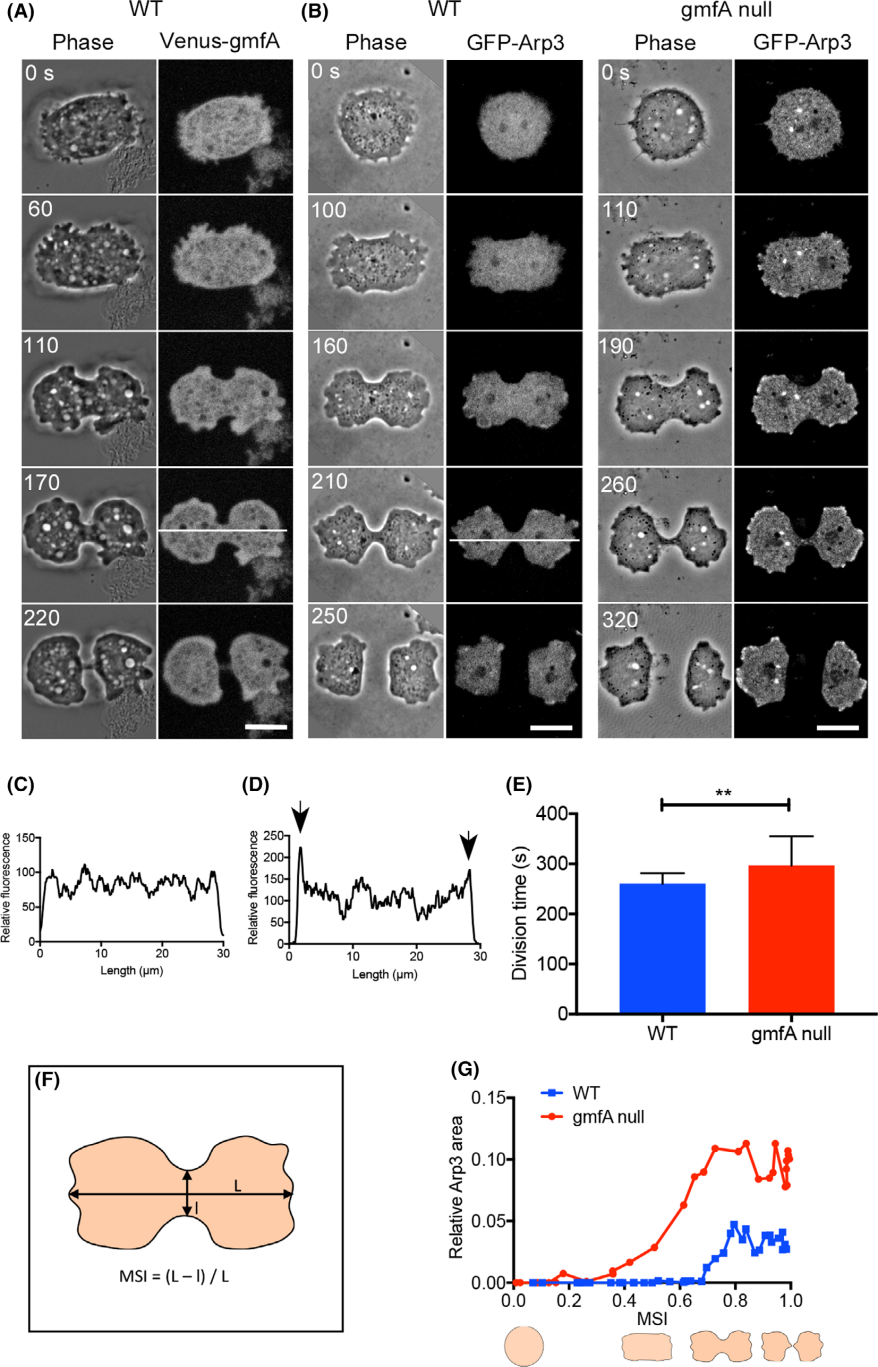
Dividing gmfA‐null cells have broader polar pseudopods. (A) A typical time course of fluorescence images of a dividing cell expressing Venus‐gmfA. Scale bars = 10 µm. (B) Typical time courses of dividing wild‐type and gmfA‐null cells expressing GFP‐Arp3. Scale bars = 10 µm. (C, D) Profiles of the fluorescence intensities on the white lines drawn on the dividing cells shown in panels A and B. Arrows indicate that GFP‐Arp3 localizes at the polar pseudopods. © A comparison of division time (from rounding to the abscission) of wild‐type and gmfA‐null cells. Data are presented as the mean ± SD (*n* > 50 for each). **P < 0.001, Student’s *t*‐test. (F) Explanation of MSI, which is calculated from the long‐axis (*L*) and short‐axis (*l*) of the cell. (G) Typical time courses of the area of polar pseudopods in wild‐type and gmfA‐null cells.

Figure 4A shows a typical time course of fluorescence images of a dividing cell expressing Venus‐gmfA. Figure 4B shows the typical time courses of dividing wild‐type and gmfA‐null cells expressing GFP‐Arp3. GmfA appeared to localize at the polar pseudopods, similar to actin localization [30, 31]. However, the profiles of the fluorescence intensities along the white lines drawn in the dividing cells in Fig. 4A suggest that gmfA does not localize at the polar pseudopods (Fig. 4C). In contrast, GFP‐Arp3 was found to localize at the polar pseudopods based on the analysis of line scanning (Fig. 4B,D). Figure 4E shows a comparison of division time (from cell rounding to abscission) in wild‐type and gmfA‐null cells. The mutant cells took a significantly longer time for cell division than the wild‐type cells (Fig. 4E), suggesting again that gmfA‐null cells had a defective cytokinesis.

GmfA‐null cells appeared to have relatively broader polar pseudopods than the wild‐type cells (Fig. 4B, right panel). Next, we examined the area of the polar pseudopods during cell division. Because the division time varied between cells, we used the mitosis stage index (MSI; calculated using long and short axes) to normalize the cell division time [32]. When the MSI was 0, the cell shape was round, corresponding to the metaphase; when the MSI was 1, the cell separated into two daughter cells (Fig. 4F). Figure 4G shows a typical time course of the area of polar pseudopods in wild‐type and gmfA‐null cells, indicating that gmfA‐null cells initiate extension at a much earlier mitotic stage with much broader polar pseudopods as compared to the wild‐type cells.

### Role of gmfA in *Dictyostelium* development

**Fig. 5:**
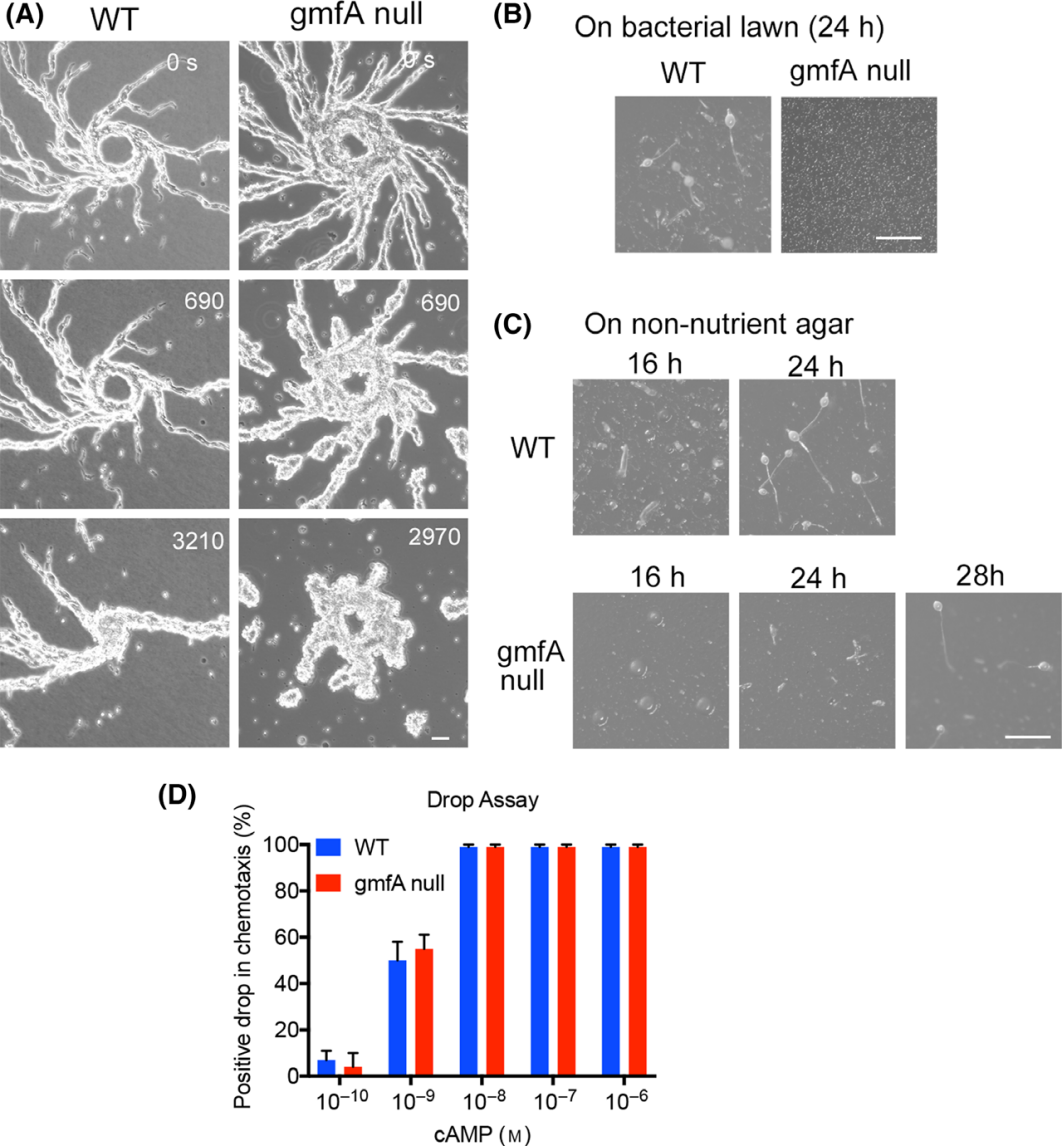
Role of gmfA in *Dictyostelium* development. (A) Typical time courses of aggregation in wild‐type and gmfA‐null cells. Scale bar = 10 µm. (B) Development of wild‐type and gmfA‐null cells on the bacterial lawn. Scale bar = 1 mm. (C) Development of wild‐type and gmfA‐null cells on the non‐nutrient agar plate. Scale bar = 1 mm. (D) Results of chemotaxis drop assay in wild‐type and gmfA‐null cells. Positive drops were counted at various cAMP concentrations (*n* = 30 for each).

*Dictyostelium* has not only a single‐cell stage but also a multicellular stage. After starvation, individual cells aggregate to form streams to the aggregation center. Aggregation is mediated by the chemotaxis of cells toward cAMP excreted from the aggregation centers. Aggregation results in the formation of a multicellular organism and finally forms fruiting bodies consisting of spores and stalks. Figure 5A shows typical time courses of aggregation in wild‐type and gmfA‐null cells. Although gmfA‐null cells initially formed aggregation streams, the streams were frequently broken when compared to wild‐type cells, suggesting that the cell–cell connection was weaker in the mutant cells than that in the wild‐type cells. In the streams, the mutant cells showed elongated shape comparable to wide‐type cells.

*Dictyostelium* cells can be cultured either in the presence of bacteria or in a nutrient medium. Interestingly, when gmfA‐null cells were cultured in the presence of bacteria, they formed neither an aggregation stream nor fruiting bodies, as opposed to wild‐type cells (Fig. 5B). As long as we observed the growth of cellular plaques in the bacterial loan, the growth of gmfA‐null cells was comparable to that of wild‐type cells. On the other hand, when they were cultured in a nutrient medium and then placed on a non‐nutrient agar plate in the absence of bacteria, they formed normal fruiting bodies, although it took a much longer time (Fig. 5C).

Drop assays were conducted to assess the chemotaxis ability. In this assay, a drop of aggregation‐competent cells was placed next to a drop of cAMP on a hydrophobic agar surface. As cAMP diffused through the agar, it formed a gradient, making the cells migrate toward it. Since the cells were not able to cross the boundary of their drop, they accumulate at the edge facing the cAMP. The extent of cell accumulation was scored over time and compared between the cell lines. Figure 5D shows the results of the drop assay, suggesting that gmfA‐null cells showed normal chemotaxis with similar sensitivity when compared to the wild‐type cells.

## Discussion

‘Dendritic nucleation model’ of actin polymerization is a widely accepted scheme of how lamellipodia or pseudopods extend [19, 33]. In this model, the Arp2/3 complex binds to the side of an existing actin 'mother filament' and nucleates the formation of a ‘daughter filament’ branch. In this manner, the Arp2/3 complex forms arborized or ‘dendritic’ actin filament networks, which push the cell membrane to extend pseudopods. Such branched actin filaments have also been visualized in tomograms of rapidly frozen *Dictyostelium* cells [34].

Gmf1, a homolog of GMF from *Saccharomyces cerevisiae*, catalyzes the debranching of daughter filaments by dissociation at branch sites *in vitro* [35]. In the present study, wild‐type cells extended multiple pseudopods with repeated extension and retraction, but gmfA‐null cells constantly extended a single broader pseudopod without retracting. It is plausible that in the gmfA‐null cells, the Arp2/3 complex may excessively enhance the nucleation of actin filaments at the leading edge, thereby causing multiple pseudopods to fuse with each other; the retraction of pseudopods may be hampered by the loss of debranching by the Arp2/3 complex, resulting in the constant extension of a broader pseudopod.

GmfA seemingly localized at the pseudopods in migrating and dividing cells, but a line scanning of fluorescence intensities suggested that it did not specifically localize at the pseudopods. Since pseudopods have virtually no organelles, such as ribosomes, endoplasmic reticulum, and mitochondria, the exclusion volume by these organelles is much smaller in pseudopods than in other cytoplasmic regions, which gives an impression that gmfA localizes at the pseudopods, as also has been discussed previously [36].

Glia maturation factor has been found at the leading edges in some cases, but is not as prominent in other cases [5, 11, 27]. Since GMF is considered to facilitate actin disassembly, it is plausible that GMF associates with the actin networks in the pseudopod for a very short duration; this may be especially true in fast‐moving cells, such as *Dictyostelium* cells, wherein actin filaments decline in 3 s after the pseudopods stop their extension [37]. However, we did not detect any increase in fluorescence signals of gmfA, even in short‐interval imaging during the retraction of the pseudopods.

GmfA‐null cells showed a keratocyte‐like shape and movement. AmiB (aggregation minus B)‐null *Dictyostelium* cells are also known to show keratocyte‐like shape and movement [38]. This gene is required for the aggregation process of *Dictyostelium*, through induction of gene expression, including that of adenylyl cyclase [39]. However, the molecular mechanism for converting the cells to a keratocyte‐like shape has not been revealed. Notably, phospholipase D is known to be essential for the maintenance of keratocyte‐like migration in NBT‐II cells, which is a mammalian epithelial cancer cell line [40]. Phospholipase D is an enzyme that hydrolyzes phosphatidylcholine to produce signal molecules, phosphatidic acid, and soluble choline.

In a simulation model for cell locomotion, which includes two parameters, namely actin assembly rate and threshold for actin polymerization (concentration of factors to start the disassembly of actin structure), when the former parameter is larger than the latter, the mathematical cell model migrates in an amoeboid‐like fashion. On the other hand, when the latter parameter exceeds the former, the cell model migrates with higher persistence in a keratocyte‐like fashion with a broader pseudopod [41]. Among the actin disassembly factors, deletion of coronin does not result in a keratocyte‐like shape [42]. Cofilin is difficult to delete because it may be lethal [43]. Therefore, it is plausible that gmfA is the main regulator of actin disassembly, as shown in the above‐mentioned simulation model.

Previously, knockdown of GMFγ has been reported in human T lymphocytes and neutrophils. The GMFγ‐knockdown T lymphocytes showed little or no net migration due to an increase in cell‐substratum binding strength [27]. The GMFγ‐knockdown neutrophils extended multiple small pseudopods, thereby losing their polarity [26]. Consequently, both T lymphocytes and neutrophils had chemotaxis defects. These observations are inconsistent with the present observations in *Dictyostelium* cells; gmfA‐null cells showed chemotaxis with cell velocities comparable to those of wild‐type cells, although their cell‐substratum adhesion became stronger. The discrepancies may be explained by use of different model organisms; however, *Dictyostelium* cells have many common and similar actin‐regulating proteins and signal proteins to those found in mammalian cells. An alternative reason may be the difference between the knockdown and knockout of GMF genes. About 10–30% of GMF, which is left in the knockdown cells, may alter the actin dynamics because the actin structures are based on the dynamic equilibrium or balance maintained by many actin‐regulating proteins.

GmfA‐null cells showed a defect of growth. This defect may be explained by their defect of cytokinesis. The multinucleation in approximately 30% of cell population should reduce the growth rate in suspension. However, the multinucleation was not prominent in the surface culture. Since the actin polymerization is also required for macro‐pinocytosis, which may also cause the defect of growth.

The defect of cytokinesis was observed especially in suspension culture. *Dictyostelium* cells have multiple modes of cytokinesis, including contractile ring‐dependent and ring‐independent modes [44, 45]. One of the latter modes is involved in the traction force of both migrating daughter halves by exerting to the substratum [32, 46]. Since the defects were not severe on the surface, the contractile ring‐dependent mode may be hampered and traction‐mediated cytokinesis may not be hampered to the same extent by the deletion of gmfA. Arp3 localization at the polar pseudopods and not at the cleavage furrow does not seem to support this idea, but its localizations may indirectly affect furrow constriction. For example, inhibition of the Arp2/3 complex affects constriction of the contractile ring, even though it does not localize at the cleavage furrow [12].

Interestingly, gmfA‐null cells could form fruiting bodies in the absence of bacteria, but could neither aggregate nor form fruiting bodies when cultured with accompanying bacteria. We have previously reported that when grown in the presence of bacteria, mfeA (fatty acid oxidation enzyme)‐null cells also could not aggregate, although they could aggregate in the absence of bacteria [47]. When bacterially grown, mfeA‐null cells accumulated excess cyclopropane fatty acids, which may affect the normal relay of cAMP and cell–cell contact. How gmfA is connected to fatty acid oxidation and the underlying signal relay need to be examined in the future.

The activities of GMF are regulated by its phosphorylation [5, 48, 49] and gmfA also seems to have putative phosphorylation sites. The regulation of gmfA and identification of a responsible kinase also needs to be addressed in the future.

## Materials and methods

### Cell culture and development

*Dictyostelium discoideum* wild‐type (AX2) cells and gmfA‐null cells were cultured in HL5 medium (1.3% bacteriological peptone, 0.75% yeast extract, 85.5 mm d‐glucose, 3.5 mm Na_2_HPO_4_, and 3.5 mm KH_2_PO_4_, pH 6.4) at 22 °C. These cells were cultured in conical flasks with a reciprocal shaker at 150 r.p.m. or in plastic dishes. To initiate development, the nutrient medium was exchanged with 15 mm Na/K phosphate buffer (PB), and the cells were incubated in the same solution for 5–6 h (pre‐aggregation stage). For analysis of development, AX2 and mutant cells that were cultured in HL5 or on 5LP plates (1.5% agar containing 0.5% lactose and 0.5% peptone) were transferred onto a 1.5% agar PB plate at a surface density of 1 × 10^6^ cells·cm^−2^ and incubated at 22 °C. For analyses of bacterially grown cells, cells were grown with *Klebsiella aerogenes* on 5LP plates.

### Plasmid construction and transformation

A gene‐targeting construct for generating *gmf*A‐null cells was prepared by inserting the blasticidin S resistance gene expression cassette (*bsr*) into the coding region using the fusion PCR technique [50]. Briefly, the 5′‐flanking region of the construct was amplified using two primers: 5′‐ TGTTGAACAACACTTGAAGC ‐3′ and 5′‐ GTAATCATGGTCATAGCTGTTTCCTGCAGCTGGTAAAGTGCAGGTGGCC‐3′. The 3′‐flanking region of the construct was amplified with following primers: 5′‐ TGCTAATCTATCAGATATTG ‐3′ and 5′‐CACTGGCCGTCGTTTTACAACGTCGACAATTAGCTTTCTTCAAATAG‐3′. The *bsr* cassette in the multi‐cloning site of pUCBsr ΔBam [51] was amplified using the primer pair 5′‐CTGCAGGAAACAGCTATGACCATGATTAC‐3′ and 5′‐GTCGACGTTGTAAAACGACGGCCAGTG‐3′, both of which are complementary to the above‐mentioned two underlined regions, respectively. The three amplified fragments were subjected to fusion PCR to produce the required gene‐targeting construct. The gene‐targeting constructs were cloned using TOPO TA cloning kit for sequencing (Invitrogen Corp., Carlsbad, CA, USA).

Full‐length *gmf*A was amplified with a cDNA mixture prepared from aggregation‐stage mRNA using an oligo‐dT primer and verified by sequencing. To construct the overexpression and fluorescent protein fusion vectors, the full‐length *gmf*A cDNA was fused with the HA‐tag sequence (YPYDVPDYA) at the 5′‐region by PCR and cloned into the cloning site of the expression vector pHK12neo‐N‐Venus [52]. The expression vector of GFP‐Arp3 was obtained from NBRP Nenkin [28].

Expression vectors containing HA‐tag‐N‐Venus‐gmfA and GFP‐Arp3 were transformed into cells by electroporation or laserporation, as described previously [50, 53, 54]. Positive cells were selected using 10 µg·mL^−1^ G418 (Wako, Osaka, Japan). After transformation with the knockout constructs, the cells were selected using HL5 medium containing 10 μg·mL^−1^ blasticidin S hydrochloride (Wako).

The phylogenetic tree was generated using mega software (https://www.megasoftware.net) after alignment with clustalw software (http://www.clustal.org).

### Microscopy

To observe cell nuclei, fixed cells were stained with DAPI (4′,6‐diamidino‐2′‐phenylindole dihydrochloride, Sigma‐Aldrich, Tokyo, Japan) as described previously [55]. Fluorescence images of DAPI‐stained cells were acquired using a fluorescence microscope (TE 300, Nikon, Tokyo, Japan) equipped with a regular UV filter set.

To observe the fluorescence of live cells expressing GFP tagged proteins, after cells were attached to the surface of the glass‐bottom chamber, they were mildly pressed with an agarose block as described previously [30, 56]. Fluorescence images were acquired using a confocal microscope (LSM510 Meta, Zeiss, Oberkochen, Germany) with a 60× objective at appropriate time intervals.

To normalize the cell division stages, the mitosis stage index (MSI) was used [32]. MSI was calculated from the long axis (*L*) and short axis (*l*). The short axis represents furrow width. The MSI was calculated using the following formula:

MSI=(L‐1)/L.

When the MSI was 0, the cell shape was round; when the MSI was 1, cell division was complete.

The images during the developmental time course were captured using a digital stereomicroscope (SZX12; Olympus, Tokyo, Japan).

### Chemotaxis assay

AX2 and gmfA‐null cells were starved for 8 h on a non‐nutrient agar plate and harvested. The cells were then resuspended in PB at a density of 1 × 10^7^ cells·mL^−1^. Cell droplets were spotted onto a PB agar plate containing 3 mm caffeine close to the cAMP droplets on a non‐nutrient agar plate. Chemotaxis toward various doses of cAMP was examined (30 cell droplets were examined for each cAMP concentration). The chemotaxis index was defined as the ratio of cells that moved toward the chemotactic source and those that moved in the opposite direction. The results are shown as the percentage of the number of positive droplets that reacted in the direction toward cAMP to the total number of droplets.

### Adhesion assay

The cells were allowed to adhere to the bottom surface of the plastic dishes for 30 min. Ten minutes after shaking by a reciprocal shaker at various speeds (r.p.m.), the detached cells were counted.

### Image processing

Cell tracking was performed using a plug‐in ‘manual tracking’ in imagej software (http://rsbweb.nih.gov/ij) and then by using a plug‐in ‘chemotaxis tool’ to create the cell trajectory graphs. The average velocity and directional persistence were generated using the latter plug‐in. The directional persistence was calculated by dividing the distance between the origin and endpoints by the actual distance along the cell trajectory.

The analyses for pseudopod areas, cell shape index, and line scanning of fluorescence intensities were also conducted using imagej software.

### Statistical analysis

Statistical analysis was conducted using graphpad prism 7 (GraphPad Software, Inc., San Diego, CA, USA) (https://www.graphpad.com). Data are presented as mean ± SD and were analyzed using unpaired two‐tailed Student’s *t*‐test.

## Conflict of interest

The authors declare no conflict of interest.

## Author contributions

KF, KN, and HK were involved in the experimental work and data analysis. KN, HK, and SY were involved in the project planning and data analysis. KF, KN, HK, and SY wrote the manuscript.

## Supporting information


**Movie S1**. Migration of wild‐type cells.

**Movie S2**. Migration of gmfA null cells.

**Movie S3**. Migration of a gmfA null cell expressing Venus‐gmfA.

## Data Availability

All relevant data are available from the authors on reasonable request.
